# Development of Nanoparticle Adaptation Phenomena in Acinetobacter baumannii: Physiological Change and Defense Response

**DOI:** 10.1128/spectrum.02857-22

**Published:** 2023-01-10

**Authors:** Oliver McNeilly, Riti Mann, Max Laurence Cummins, Steven P. Djordjevic, Mehrad Hamidian, Cindy Gunawan

**Affiliations:** a Australian Institute of Microbiology and Infection, University of Technology Sydney, Broadway, New South Wales, Australia; b Australian Centre for Genomic Epidemiological Microbiology, University of Technology Sydney, Broadway, New South Wales, Australia; c School of Chemical Engineering, University of New South Wales, Sydney, New South Wales, Australia; CCG-UNAM

**Keywords:** *Acinetobacter baumannii*, mutation, resistance, silver nanoparticles

## Abstract

The present work describes the evolution of a resistance phenotype to a multitargeting antimicrobial agent, namely, silver nanoparticles (nanosilver; NAg), in the globally prevalent bacterial pathogen Acinetobacter baumannii. The Gram-negative bacterium has recently been listed as a critical priority pathogen requiring novel treatment options by the World Health Organization. Through prolonged exposure to the important antimicrobial nanoparticle, the bacterium developed mutations in genes that encode the protein subunits of organelle structures that are involved in cell-to-surface attachment as well as in a cell envelope capsular polysaccharide synthesis-related gene. These mutations are potentially correlated with stable physiological changes in the biofilm growth behavior and with an evident protective effect against oxidative stress, most likely as a feature of toxicity defense. We further report a different adaptation response of A. baumannii to the cationic form of silver (Ag^+^). The bacterium developed a tolerance phenotype to Ag^+^, which was correlated with an indicative surge in respiratory activity and changes in cell morphology, of which these are reported characteristics of tolerant bacterial populations. The findings regarding adaptation phenomena to NAg highlight the risks of the long-term use of the nanoparticle on a priority pathogen. The findings urge the implementation of strategies to overcome bacterial NAg adaptation, to better elucidate the toxicity mechanisms of the nanoparticle, and preserve the efficacy of the potent alternative antimicrobial agent in this era of antimicrobial resistance.

**IMPORTANCE** Several recent studies have reported on the development of bacterial resistance to broad-spectrum antimicrobial silver nanoparticles (nanosilver; NAg). NAg is currently one of the most important alternative antimicrobial agents. However, no studies have yet established whether Acinetobacter baumannii, a globally prevalent nosocomial pathogen, can develop resistance to the nanoparticle. The study herein describes how a model strain of A. baumannii with no inherent silver resistance determinants developed resistance to NAg, following prolonged exposure. The stable physiological changes are correlated with mutations detected in the bacterium genome. These mutations render the bacterium capable of proliferating at a toxic NAg concentration. It was also found that A. baumannii developed a “slower-to-kill” tolerance trait to Ag^+^, which highlights the unique antimicrobial activities between the nanoparticulate and the ionic forms of silver. Despite the proven efficacy of NAg, the observation of NAg resistance in A. baumannii emphasises the potential risks of the repeated overuse of this agent on a priority pathogen.

## INTRODUCTION

The world is in the midst of an antimicrobial resistance (AMR) emergency, wherein many antibiotic drugs no longer have an effect against bacterial pathogens (1, 2). The widespread overuse of antibiotics across industrial and medical settings, along with a significant withdrawal in financial investment into antibiotic research and development, have ultimately led to this global health crisis (3–5). Acinetobacter baumannii is a Gram-negative coccobacillus bacterium, and it is a member of the ESKAPE group, which includes the leading nosocomial (hospital-related) antibiotic-resistant pathogens (Enterococcus faecium, Staphylococcus aureus, Klebsiella pneumoniae, Acinetobacter baumannii, Pseudomonas aeruginosa, and Enterobacter spp.) (6–8). The World Health Organization (WHO) has recently listed carbapenem-resistant A. baumannii (CRAb) as a critical level priority pathogen (priority level one) that requires the immediate development of new and effective treatment options (2, 9). It is difficult to control CRAb infections, given that, in addition to them having resistance to carbapenems (considered last line antibiotics), these strains are also resistant to nearly all other antibiotics. The global distribution of CRAb strains is mainly due to the spread of two clonal sequence types, namely, ST1 and ST2, also known as global clone 1 and 2, respectively (10, 11). In 2019, there were 132,000 global deaths attributed to multidrug resistant (MDR) A. baumannii, 37,700 of which were carbapenem resistance-related deaths (12). The bacterium has an incredible innate ability to acquire resistance to many antibiotics through the uptake of mobile genetic elements (e.g., transposons and plasmids) that carry AMR genes (7, 13). Carbapenem resistance in A. baumannii is primarily due to the horizontal acquisition of genes encoding carbapenem-hydrolysing oxacillinases (e.g., *oxa23*, *oxa24*, and *oxa58*) (14, 15).

With the global spread of AMR, an increasing number of research efforts have been dedicated toward the development of alternative antimicrobials, particularly those with multitargeting mechanisms. Silver nanoparticles (herein referred to as nanosilver or NAg) are ultrafine, less than 100 nm particulates of metallic silver (Ag^0^) or silver oxide (Ag_2_O). NAg is among the most commercialized alternative antimicrobial agents, due to its potent and broad-spectrum antimicrobial properties (16, 17). The nanoparticles have been used in medical devices, such as wound dressings, catheters, and prosthetics, to treat and prevent infections (18, 19). NAg has also been incorporated in a vast array of everyday products and appliances, including personal care products, clothing, textiles, washing machines, fridges, and even baby products, in an attempt to impede microbial growth (18, 20, 21). This increasingly indiscriminate use of the nanoparticle has brought concern to the scientific community as to whether, just like in the case of antibiotics, bacteria could develop resistance to NAg.

While bacterial resistance to cationic silver (Ag^+^) has been recognized for several decades, (22) it was a common perception that resistance to NAg was unlikely, due to the multitargeting antimicrobial mechanisms of the nanoparticles (18, 23). However, contradicting this notion, there has been growing evidence over the past decade regarding the development of adaptation phenomena to NAg, and this evidence has been observed in a number of bacterial species, including those of clinical significance, such as Escherichia coli, Pseudomonas aeruginosa, and Staphylococcus aureus (23–28). In the case of A. baumannii, studies have previously reported mechanisms of Ag^+^ resistance in the bacterium, including the acquisition of mobile genetic elements (plasmids) that harbor the well-known Sil Ag^+^ efflux system (encoded by the *sil* operon *silESRCFBAGP*) (29–31). Nevertheless, to date, there has been no reported evidence of NAg resistance in A. baumannii or in any other Acinetobacter species, in fact, and little is known regarding the bacterial species’ interaction with the nanoparticle (32).

To address this knowledge gap, the present work aims to study the toxicological and subsequent adaptation responses of A. baumannii to long-term NAg exposure. More specifically, this work seeks to gain insights on how the bacterium evolves physiological changes as defense mechanisms against the toxicity of the nanoparticles in the absence of the Sil efflux system. The latter is to determine whether A. baumannii has the intrinsic ability to develop resistance to NAg without the acquired silver efflux system. Herein, we observe the development of stable adaptation characteristics in A. baumannii (reference strain ATCC 19606; GenBank accession number CP045110; with no presence of *sil* genes) (33) in response to prolonged NAg exposure. The bacterium evolved single nucleotide gene mutations, with evidence suggesting links between these mutations and the observed changes in the ability of the bacterium to form biofilms, as well as, indicatively, to its protective mechanisms against NAg-induced cellular oxidative stress. The work also found that A. baumannii developed a different adaptation response to Ag^+^ with distinct physiological changes, thereby highlighting the unique microbiological activities of the nanoparticulate versus ionic forms of silver. A parallel evolutionary study with nalidixic acid (Nx) was also included to compare the extent of resistance development with a single-target model antibiotic.

## RESULTS AND DISCUSSION

### Toxicology and the evolution of adaptation responses of A. baumannii to NAg, Ag^+^, and Nx.

**FIG 1:**
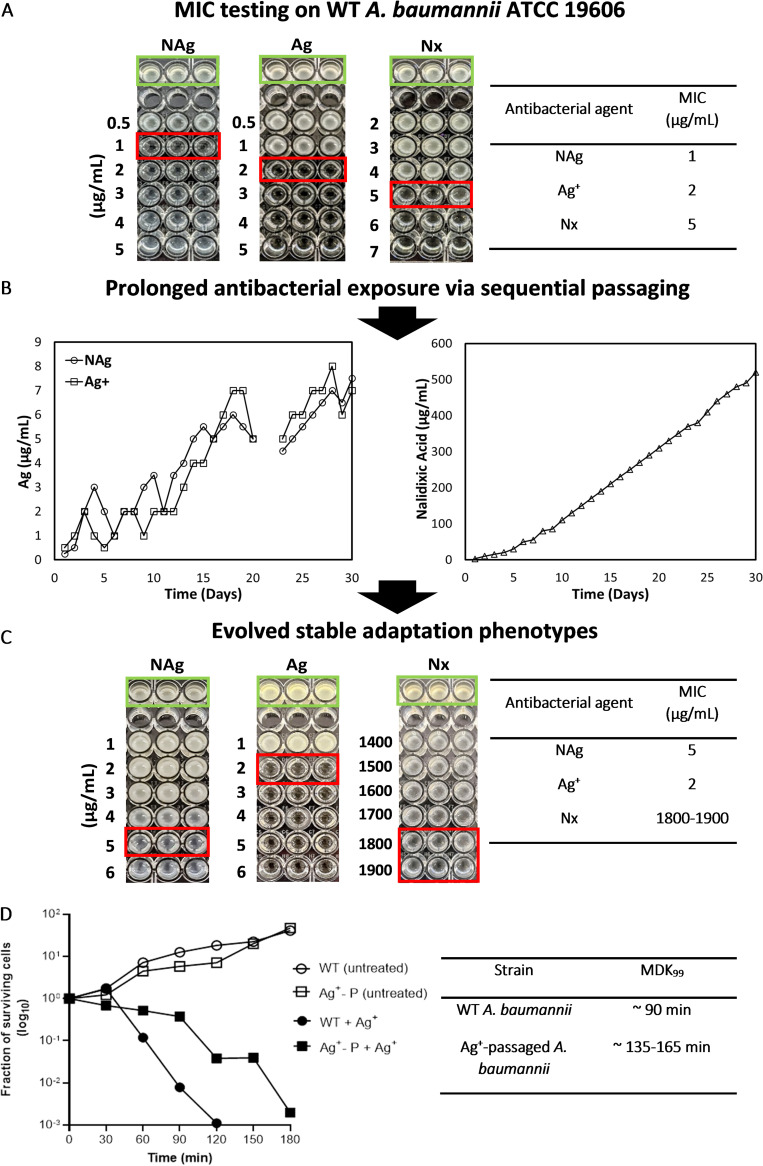
Evolution of adaptation phenotypes to NAg, Ag^+^, and Nx in A. baumannii. (A) Determination of the initial MICs of NAg, Ag^+^, and Nx on wild-type (WT) A. baumannii. The experiments were performed in three biological replicates (independent bacterial inoculum from three individual colony isolates per antibacterial agent, each with three technical replicates). One biological replicate per treatment system is shown (green outline, cell-only control; red outline, MIC point; media-only control not shown). (B) Sequential passaging experiments (subculturing every 24 h) showing the progressive shifts in the highest concentrations of NAg, Ag^+^, and Nx at which the bacterium could proliferate (over 30 days). The experiments were performed in two biological replicates (for each agent) and included a cell-only control passaged culture (no antimicrobial agent) for each condition, including three technical replicates. Note that the 3-day passaging gap in the NAg/Ag^+^ exposure experiments were due to a laboratory closure. See Fig. S1 for the passaging results of the second biological replicate. (C) The NAg, Ag^+^, and Nx MIC reassessment on the passaged A. baumannii cultures (isolates obtained at the endpoint of passage). Shown here are the results of the first biological replicate passaging culture. See Fig. S1 for the MIC results of the second biological passaged replicate. (D) The killing kinetics of WT and Ag^+^-passaged (Ag^+^-P) A. baumannii when exposed to a 1.5 × MIC Ag^+^ dose to determine the minimum duration of killing for 99% (MDK_99_) of the cell populations. The graph plot shows one technical time-kill assay replicate of the first biological passaged replicate (for the Ag^+^-P strain). See Fig. S1 for all of the other biological and technical replicates.

To investigate the adaptation responses of A. baumannii to NAg, Ag^+^ (supplied as AgNO_3_) and the model antibiotic Nx, the minimum inhibitory concentration (MIC) of each antibacterial agent was first determined against strain ATCC 19606. From our dose-dependent growth inhibition studies, the MIC was observed to be 1 μg/mL for NAg and 2 μg/mL for Ag^+^ (Fig. 1A). NAg and Ag^+^ are known to target cell envelopes, which, in the case of Gram-negative bacteria, such as A. baumannii, involves damage to phospholipid moieties in their outer and inner membranes (34). The targeting of the inner membrane is thought to disrupt bacterial respiratory components (embedded within the membrane). For instance, Ag^+^ can form complexes with electron donor groups in proteins (e.g., thiol and amine groups), including those that are present in the respiratory enzyme NADH dehydrogenase (17, 35–38). This membrane-targeting activity likely causes electron leakage from respiratory components, which can result in the generation of reactive oxygen species (ROS) that further damage biomolecules, including DNA, lipids, and other proteins (17, 34). Although there are some similarities, studies have shown that NAg exerts unique antimicrobial mechanisms, compared to Ag^+^. In an aqueous environment, NAg can undergo oxidative dissolution and release Ag^+^ ions that can interact with various moieties (including halides, sulfides, and organics) and transform into other distinct silver species, each with its own toxicity (33, 35). Previous studies have shown that Ag^+^, in general, exerts a higher extent of antibacterial activity, compared to NAg, as the activity of the nanoparticle is determined by its physicochemical characteristics, such as size, shape, and surface charge (35, 39–41). Our results, however, are indeed consistent with the reported NAg MICs of 0.39 to 2.5 μg/mL on A. baumannii (42, 43), as well as with one Ag^+^ study that reported a MIC of 4 μg/mL (44). Nx is a single-target fluoroquinolone antibiotic that inactivates the DNA gyrase subunit GyrA, which is an enzyme that plays a role in the unwinding of DNA during cell replication (45). Herein, we observed a 5 μg/mL MIC for Nx (Fig. 1A).

Next, the ability of A. baumannii to evolve adaptation characteristics to NAg, Ag^+^, and Nx was investigated. The bacterium was exposed to progressively increasing concentrations of each agent through sequential passaging (subculturing) every 24 h over 30 days. The prolonged exposures saw progressive shifts in the highest antimicrobial doses at which the bacterium could proliferate. On day 1, A. baumannii was only able to grow at maximum concentrations of 0.25 and 0.5 μg/mL of NAg and Ag^+^, respectively. At the conclusion of the exposure courses, the bacteria could proliferate at 7 μg/mL of NAg (day 30) and 8 μg/mL of Ag^+^ (day 28) (Fig. 1B). For Nx, the bacterium was proliferating at an extremely high concentration of 520 μg/mL by day 30, up from 2.5 μg/mL on day 1 (Fig. 1B). To assess the development of stable resistance traits, the MICs of each agent were assessed against the sequentially passaged cultures. It was found that the MIC of NAg had increased to 4 to 5 μg/mL (4 to 5× MIC of that observed for the wild-type [WT] strain) and to 1,800 to 1,900 μg/mL for Nx (360 to 380× MIC of the WT), whereas the MIC of Ag^+^ was unaffected, remaining as that of the WT strain at 2 μg/mL (Fig. 1C; Fig. S1B). Herein, we can conclude that A. baumannii developed stable resistance to NAg, and, as expected, to Nx, due to prolonged exposure. A resistance effect develops when a bacterial population is able to proliferate at an otherwise toxic concentration of an agent (3). This effect is confirmed when there is a twofold or higher increase in the MIC of an agent on an exposed bacterial strain (46). It should be noted that the sequentially passaged bacterial isolates were cultured in antimicrobial-free media (for three days) prior to the MIC reassessment. This was to exclude any cell population with transient adaptation traits (47). A. baumannii has been known to develop resistance to Nx through single point mutations in the *gyrA* gene (48), which was also detected in the present work, following the Nx passaging experiment, as is later described (see “Genetic mutations and physiological changes in silver-adapted A. baumannii“). Exceptionally high Nx MICs (>1,000 μg/mL), as observed in this study, have been previously reported in other Nx-resistant A. baumannii strains (49, 50). The inclusion of the Nx resistance study was not only to investigate the distinct adaptation responses of A. baumannii to the antibiotic but also to validate the sequential passaging experiment. To the best of our knowledge, this is the first reported evidence of (stable) NAg resistance in A. baumannii.

Earlier studies have reported (ionic) silver resistance in the bacterium (29–31, 51). Hosny et al. detected the presence of the Ag^+^ efflux Sil system in two A. baumannii strains that were isolated from patient wounds and treated with 1% silver sulfadiazine (31). This well-established efflux mechanism is encoded by a nine-gene *sil* operon (22, 52), which is often found in mobile genetic elements, such as plasmids (32). The *silE*, *silS*, *silR*, *silC*, *silF*, *silB*, *silA*, *ORF105* (*silG*), and *silP* genes encode functional proteins in three transcriptional units (SilE, SilRS, and SilCFBAGP) (53, 54). The efflux mechanism responds to the presence of intracellular Ag^+^, binding the ions to chaperone(s) and then exporting the ions out of the cell via a membrane-bound P-type ATPase efflux (22, 41, 53, 54). Recalling the absence of Ag^+^ resistance development in our A. baumannii strain, we confirmed that there were no *sil* genes present in the genome of the bacterium. The strains isolated by Hosny et al. had likely acquired their *sil* genes through the uptake of mobile genetic elements (hence the acquisition of exogenous genes) (31). To the best of our knowledge, no endogenous presence of *sil* genes has previously been reported in the A. baumannii genome (32).

Although no increase was seen in the Ag^+^ MIC for the Ag^+^-passaged strain, the bacterial growth that was observed at increasing Ag^+^ dosages during the passaging experiment (Fig. 1B) indicated that some form of adaptation phenotype had developed and that it was potentially associated with tolerance or persistence. In contrast to resistance, tolerance is the ability of a bacterial population to survive a lethal antimicrobial exposure for a longer time (with no MIC increase) due to a slower killing rate (conferred environmentally or through mutation), whereas persistence only affects a minute fraction of a clonal bacterial population (46, 55). The slower killing effect is characterized by an increase in an agent’s minimal duration for killing (MDK) the bacterial population: 99% of the population for a tolerance effect (MDK_99_), and 99.99% of the population for a persistence effect (MDK_99.99_) (28, 46, 55). A time-kill assay was performed to determine whether the Ag^+^-passaged A. baumannii strain had developed tolerance or persistence to Ag^+^. The killing kinetics of Ag^+^ (1.5× MIC dose) on the passaged strain saw an increase in the MDK_99_ (approximately 135 to 165 min), compared to that observed for the WT strain (approximately 90 min), which signifies a tolerance trait (Fig. 1D; Fig. S1C). Presumably, this is the first reported case of Ag^+^ tolerance in A. baumannii in response to prolonged Ag^+^ exposure. Tolerances to other heavy metals have been reported in A. baumannii, with one study highlighting a link between the copper tolerance and the upregulation of copper efflux genes, both endogenous (e.g., *cueR*) and exogenous (e.g., *copRS*) (56).

We did not observe any reduction in the growth rate of the Ag^+^-tolerant (Ag^+T^) strain, which exhibited a similar growth profile to that of the WT strain (Fig. S2). This indicates that prolonged Ag^+^ exposure did not induce a “tolerance by slow-growth” effect, which is a characteristic feature that is often seen in antibiotic-tolerant strains as a means to passivate antibiotics that target actively proliferating bacteria (46, 57). Studies have also described other antimicrobial tolerance mechanisms in bacteria, including changes in biofilm growth behavior and cellular ROS-associated physiological changes (58, 59). As is shown later, our study investigated these behaviors to gain clues regarding the mechanisms that could play a role in the Ag^+^ tolerance trait.

Note that there was no cross-adaptation observed between NAg and Ag^+^. The MIC of NAg for the Ag^+^-tolerant (Ag^+T^) strain, and vice versa, the MIC of Ag^+^ for the NAg-resistant (NAg^R^) strain, were comparable to their respective MICs in the WT strain (Fig. S3). The adaptation to NAg and Ag^+^ also did not provide cross-protection to Nx. The MIC of Nx for the NAg^R^ and Ag^+T^ strains were akin to that seen with the WT strain (Fig. S3). These findings are not surprising, considering the diverse antimicrobial mechanisms of NAg, Ag^+^, and the model antibiotic.

### Genetic mutations and physiological changes in silver-adapted A. baumannii.

A comparative genomic analysis was carried out to detect potential gene mutations in the silver-adapted strains. Various unique, silver-induced single nucleotide polymorphisms (SNPs) were identified in the NAg^R^ and Ag^+T^ strains (Table 1), excluding random “background” mutations. The latter are SNPs that were seen in the cell-only passaged cultures (passaged with no antimicrobial agent), and these most likely developed due to the effect of repeated subculturing (Table S1) (23). The detected silver-induced SNPs were stable, given that the adapted strains were cultured in antimicrobial-free media for several days prior to the genome sequencing. This suggests that A. baumannii had evolved physiological changes that could contribute to the defense against silver toxicity. Mutations were indeed detected in *gyrA* in the Nx^R^ strain, further validating the sequential passaging experiment. Two point mutations (associated with a change in the protein amino acid sequence) were seen in the gene (Table 1): one nucleotide substitutional mutation (*f *= 100%, *n* = 6) that resulted in a Ser81Leu change (serine at position 81 to leucine) and another substitutional mutation (*f *= 50%, *n* = 6) that was associated with an Arg70Gly change. Studies have shown that point mutations in *gyrA* lead to conformational changes in the GyrA protein, with the most frequently reported amino acid change being Ser83Leu, which blocks Nx from accessing its active site (48, 60–62). Several more SNPs were detected in other genes in the Nx^R^ strain (Table S2), but these are most likely unrelated to the Nx resistance trait.

**TABLE 1:** Gene mutations detected in the Nx-resistant (Nx^R^; only mutations detected in the target gene *gyrA* are shown), NAg-resistant (NAg^R^), and Ag^+^-tolerant (Ag^+T^) A. baumannii strains

Strain	Mutation^*a*^	Genome pos.^*b*^	AA change	Gene	Locus tag	*f* (%)^*c*^	Protein product
Nx^R^	SUB (G > A)	3108662	Ser81Leu	*gyrA*	FQU82_02986	100	DNA gyrase subunit A
	SUB (T > C)	3108695	Arg70Gly	*gyrA*	FQU82_02986	50^*d*^	DNA gyrase subunit A
NAg^R^	SUB (G > A)	659811	Ser196Phe	*rcsC* ^*e*^	FQU82_00629	16.7^*d*^	Sensor histidine kinase RcsC
	DEL (TG > T)	2146107	Ala129fs^*f*^	*smf1-2*	FQU82_02066	33.3^*g*^	Major fimbrial subunit SMF1
	SUB (G > A)	2669300	Ala20Val	*csuB*	FQU82_02552	33.3^*g*^	Csu pili biogenesis protein CsuB
	INS (C > CATA)	2669301	Tyr19dup^*h*^	*csuB*	FQU82_02552	16.7^*d*^	Csu pili biogenesis protein CsuB
Ag^+T^	DEL (GA > G)	133861	Lys4fs^*f*^	*atr2*	FQU82_00141	100	Acyltransferase
	DEL (GA > G)	135040	Met150fs^*f*^	*gtr6*	FQU82_00142	33.3^*i*^	Glycosyltransferase
	SUB (G > A)	2753686	Gly352Arg	*mdtE*	FQU82_02642	16.7^*g*^	Multidrug resistance protein MdtE
	SUB (G > A)	3407963	Arg58Cys	*trpB*	FQU82_03235	100	Tryptophan synthase beta chain
	SUB (C > T)	3878684	Gly39Arg	*gshA*	FQU82_03694	100	Glutamate-cysteine ligase
	DEL (TG > T)	3927318	Val10fs^*f*^	*iclR*	FQU82_03739	16.7^*d*^	IclR family transcriptional regulator

a Refer to Table S2 for the complete list of mutations detected in the Nx^R^ strain. Random “background” gene mutations that were detected in the cell-only passaged control (Table S1) have been excluded.

b The position of the gene mutation in the A. baumannii genome.

c *f*, frequency of mutation (%) in the isolated strain.

d Detected in only the first biological passage replicate of the respective strain.

e The mutation to the gene was also detected in the Nx^R^ strain (Table S2).

f fs, frameshift.

g Detected in only the second biological passage replicate of the respective strain.

h dup, duplication.

i Detected in each biological passage replicate of the respective strain.

As shown in Table 1, several SNPs were detected in the NAg^R^ strain in genes related to biofilm formation. A mutation was detected in the gene *rcsC* (substitution: *f *= 16.7%, *n* = 6), which encodes the sensor histidine kinase RcsC. This protein forms part of the Rcs pathway, which regulates the synthesis of the colanic acid capsular polysaccharide (CPS), a unique cell envelope component that is highly conserved in A. baumannii spp. Studies have supported a role for the involvement of CPS in biofilm formation, that is, in maintaining cell membrane integrity during bacterial attachment to surfaces (63, 64). Next, a mutation was detected in the gene *smf1-2* (deletion: *f *= 33.3%, *n* = 6), which encodes the major fimbrial subunit SMF1 (Table 1). This peritrichous, hair-like, polymeric protein structure is used by bacteria to attach to surfaces and to help initiate biofilm formation (65). Finally, mutations (substitution: *f *= 33.3%, *n* = 6; insertion: *f *= 16.7%, *n* = 6) were detected in the gene *csuB* (Table 1). This gene encodes the Csu pili protein subunit CsuB and is part of a six-gene operon *csuA/BABCDE*, which collectively codes for the Csu type 1 chaperone-usher pili (66). Pili, like fimbriae, are also hair-like protein structures that project from the bacterial envelope. Pili are known to aid in the cell-to-cell transfer of DNA during bacterial conjugation, but reports have indicated that bacteria also use these structures to attach to surfaces for biofilm growth (66). The Csu pili is, in fact, one of the most conserved biofilm-associated protein structures in globally disseminated A. baumannii clones (67, 68).

Mutations were also detected in the genome of the Ag^+T^ strain (Table 1), and at least one of these SNPs is in a gene that has been previously linked to biofilm formation. A mutation was detected in the gene *gshA* (substitution: *f *= 100%, *n* = 6), which encodes the enzyme glutamate-cysteine ligase (GCL), which is involved in the biosynthesis of the antioxidant glutathione (GSH) (69). GSH is essential for maintaining the redox balance in Gram-negative bacteria, as it scavenges for excess ROS to prevent oxidative stress (69, 70). Interestingly, studies have recognized a link between *gshA* function and biofilm formation. For instance, Wongsaroj et al. reported a higher extent of biofilm formation in a *gshA*-knockout mutant, relative to the WT strain, in P. aeruginosa (71). The study also observed reduced motility with the mutant. The *gshA* mutation detected herein could also relate to the known oxidative stress-related toxicity of Ag^+^ (72, 73). Therefore, the mutation is hypothesized to contribute to the Ag^+^ tolerance trait, of which the phenotypic features (biofilm growth and cellular ROS response) were later investigated in the present work. Notably, we also detected mutations in other genes in the Ag^+T^ strain (Table 1) (*f *= 16.7 – 100%, *n* = 6), but these had limited correlations to the Ag^+^ toxicity defense. For example, a mutation was detected in the gene *mdtE* (substitution: *f *= 16.7%, *n* = 6), which encodes the protein MdtE, a subunit of the multidrug resistant efflux system MdtEF (74). This efflux system is reported to contribute to β-lactam (antibiotic) resistance, as well as to other biocides, such as benzalkonium, in E. coli (74). Nevertheless, there is no evidence at this stage to suggest the role of MdtE in heavy metal efflux.

Considering the potential link between the detected SNPs and the biofilm formation phenotypes, it was hypothesized that there could be changes in the biofilm growth behavior of the silver-adapted strains, possibly as a stable physiological feature that developed in response to the prolonged exposure events. Biofilms are surface-attached microbial communities. Bacterial populations in biofilms are protected by an adhesive matrix structure, known as extracellular polymeric substance (EPS), that renders the colony resilient to many external stressors, including antimicrobial exposure (75–77). We assessed the biofilm growth behavior of the NAg^R^ and Ag^+T^ strains in the absence of silver. The latter was to see whether the (hypothesized) physiological changes were stable and therefore still manifested, even without silver. An increased extent of biofilm formation has been observed in bacteria in response to external stress, including silver toxicity (28, 78, 79). Such silver-induced changes in growth activity, as far as we know, have yet to be reported in A. baumannii (32). For the biofilm-formation study, we studied the silver-adapted isolates that carry the (earlier described) specific mutations in their biofilm-associated genes. For the NAg^R^ strain, an isolate with mutations in the highly conserved biofilm-associated genes *rcsC* and *csuB* (insertional mutation for the latter gene) was selected (Table 1) (GenBank accession number JAPYLV000000000), whereas for the Ag^+T^ strain, an isolate with a mutation in *gshA* was selected (Table 1) (GenBank accession number JAPYLT000000000), considering the more established role of the gene in biofilm formation.

**FIG 2:**
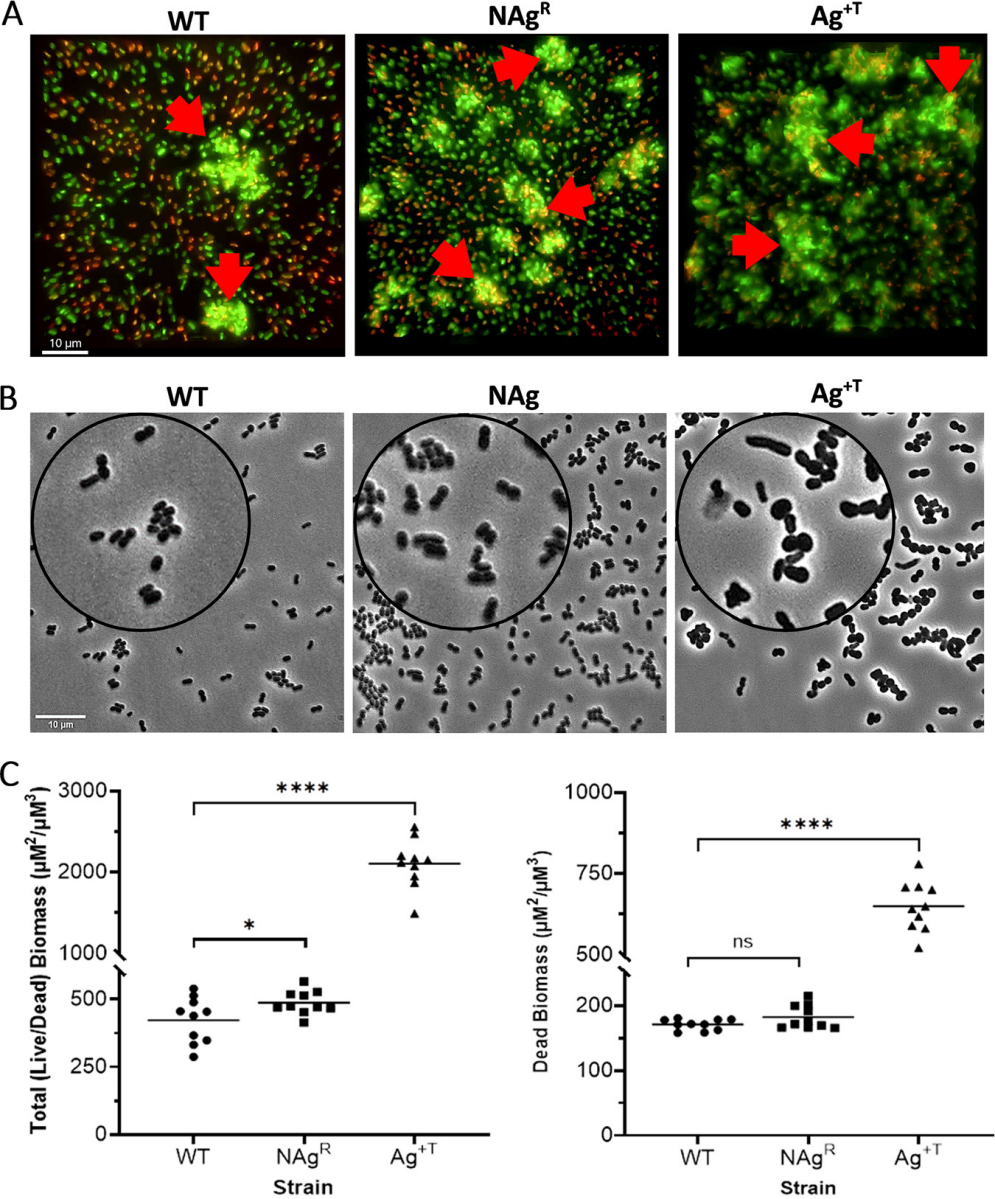
Evolved physiological changes in the NAg^R^ and Ag^+T^ A. baumannii strains. (A) Fluorescent microscopy images of live (green) and dead (red) biofilm biomass from the WT, NAg^R^, and Ag^+T^ strains. Red arrows highlight biofilm biomasses. The images are sectional Z-stacks that are compressed together into single plane 3D slices (scale bar = 10 μM). (B) Phase contrast images of planktonic WT, NAg^R^, and Ag^+T^ A. baumannii cells (scale bar = 10 μM). Magnified insets provide closer details of the cell morphology. (C) Analysis of the total biomass (live and dead, left panel) and the dead biomass (right panel) (μM^2^/μM^3^) from the fluorescence microscopy images for each strain. 10 images were captured for each biological replicate (30 images in total, with three biological replicates per strain). Each symbol (circle, square, triangle) represents the average of three analyzed images for the respective strain, with the horizontal bars representing the average of all 30 of the analyzed images per strain. Asterisks indicate statistically significant increases in the NAg^R^ and Ag^+T^ biofilm biomass, relative to those of the WT. *, *P* < 0.05; ****, *P* < 0.0001.

As shown in Fig. 2C, it was found that the long-term silver exposure had altered the biofilm-forming ability of the bacterium. Under the experimental conditions tested, the NAg^R^ strain formed approximately 15% more biofilm biomass (487 μm^2^/μm^3^ mean total [live/dead] biomass), compared to the WT strain (422 μm^2^/μm^3^ mean total biomass, *P* < 0.05). The fluorescence microscopy images (Fig. 2A, red arrows) highlight the greater extent of biofilm growth. Observations suggest that the evolved NAg-induced mutations (seen in the CPS synthesis regulatory pathway gene *rcsC* and in the Csu pili subunit gene *csuB*) could be associated with the increased biofilm growth in the strain. These mutations in the capsule envelope (CPS) synthesis and external motility organelle (pili) are hypothesized to enhance the ability of the bacterium to attach to surfaces (as described earlier) and promote further colonization. The greater biofilm-forming ability of the NAg^R^ strain would require a higher NAg dosage to control, compared to the WT strain, and it would thus serve as a resistance trait. Earlier studies have also correlated NAg resistance to cell envelope (motility) organelles. Panáček et al. reported an increased production of flagellin (a major protein component of flagella) in E. coli along with the epigenetic/nonmutational changes associated with the aggregation of NAg, which was thought to hinder nanoparticle activity (26). In another E. coli study, Stabryla et al. suggested a link between improved flagella-based motility and the development of NAg resistance. A stable resistance phenotype was only seen in the hyper-motile strain following prolonged exposure; resistance was absent in the nonmotile strain (27). It should be noted that at this stage, we are not excluding the potential role of lesser studied *A.baumannii* fimbrial subunit gene *smf1-2* in the altered biofilm-forming physiology of the NAg^R^ strain. Further molecular-focused investigations, as later described, are being conducted to better understand the role(s) of this gene (and others) in the resistance trait.

Biofilm growth studies performed on the Ag^+T^ strain yielded interesting results. Under the experimental conditions, the strain formed fivefold higher biofilm biomass (2,107 μm^2^/μm^3^ mean total biomass), relative to the WT strain (*P* < 0.0001) (Fig. 2C). The observations suggest a potential link between the *gshA* mutation in the tolerant strain to its increased biofilm growth. The Ag^+^-induced mutation in the GSH synthesis gene *gshA* is thought to decrease bacterial motility. The latter phenotype, as earlier mentioned, has been reported in a (P. aeruginosa) *gshA*-knockout mutant (Δ*gshA*), with the mutant also forming more biofilm than did the WT strain. However, the exact link between motility and the changes in biofilm growth behavior is still unclear (71). Herein observed, the greater biofilm-forming ability of the Ag^+T^ strain may require a longer time to control, compared to that of the WT strain. Hence, it could correspond to the tolerance effect.

Further examination revealed notable morphological changes in the Ag^+T^ strain that were not seen with the NAg^R^ strain (Fig. 2B). In comparison to the approximately 1 μm coccobacillus (length) of the WT strain, many Ag^+T^ cells were engorged with irregular shapes, whereas others appeared to elongate to more than twofold the length of the WT cells. Research inquiries have reported morphological changes in bacteria in response to antibacterial treatment (35, 80, 81). One study described the shortening and lengthening of E. coli cells following prolonged Ag^+^ exposure (82). This oscillation in cell shape appeared to associate with a suppression in metabolic activity (similar to tolerant/persistent behavior), which seemed to protect the bacterium from Ag^+^ toxicity (82). In A. baumannii, a number of antibiotic-adapted strains have also been reported to undergo morphological changes (81). Therefore, it is reasonable to suggest that the morphological changes seen with the Ag^+T^ strain are contributing factors to the bacterium tolerance response to the ions.

Taken together, the SNP evidence indicates that A. baumannii evolved the gene mutations in response to prolonged NAg (and Ag^+^) exposure. A number of these mutations appear to correlate with the increased biofilm-forming ability seen with both silver-adapted strains. The stable physiological changes most likely contribute to the bacterium adaptation behavior, that is, the “harder-to-kill” resistance trait in the case of NAg and the “slower-to-kill” tolerance trait for Ag^+^. The latter adaptation effect also seems to involve morphological changes in the bacterium. Finally, it is noteworthy to mention that these SNPs have been validated through the resequencing of the polymerase chain reaction (PCR)-amplified genes of interest. Next, the defense responses of the silver-adapted strains, which are exhibited when in the presence of silver, were studied.

### Cellular ROS-related defense in silver-adapted A. baumannii.

**FIG 3:**
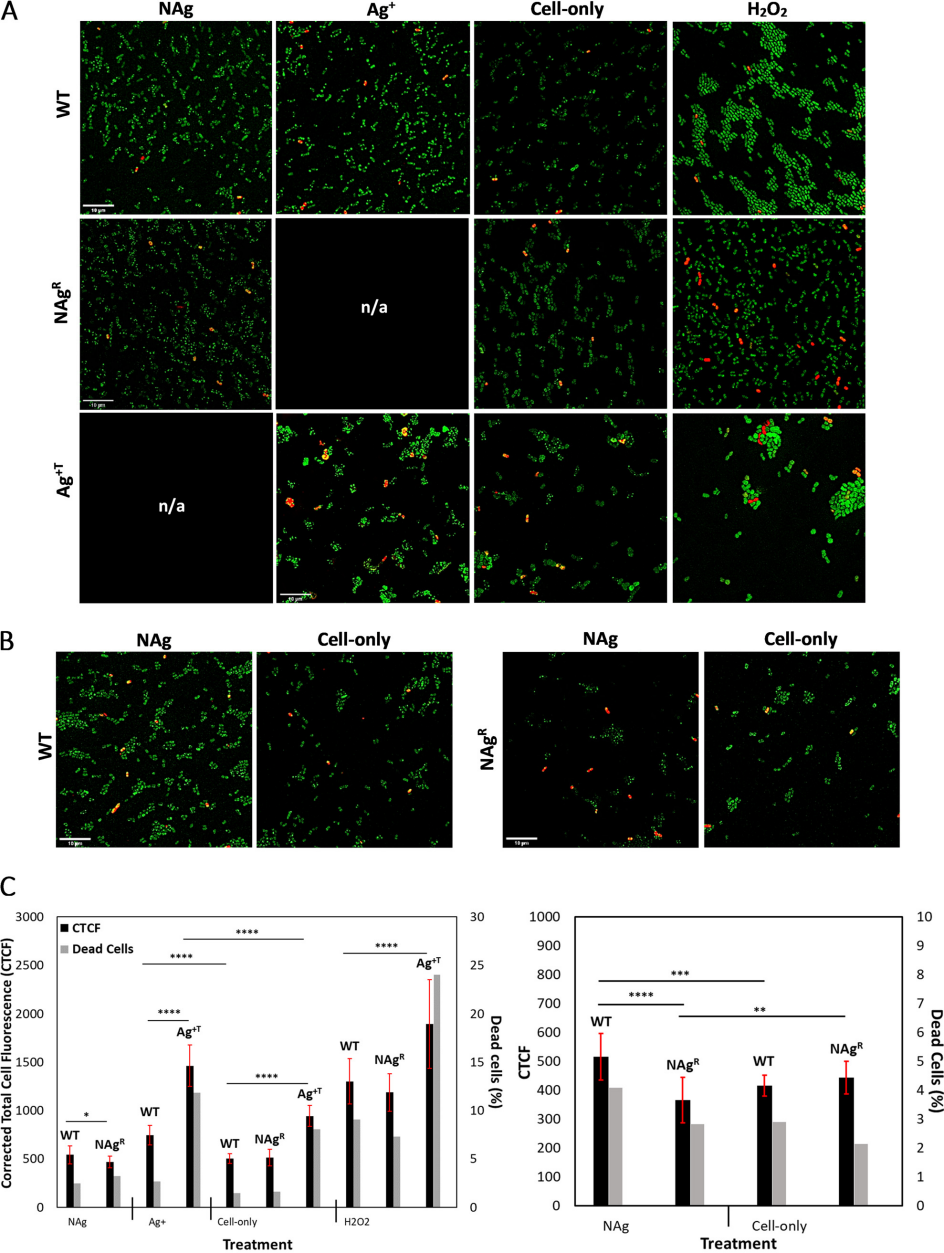
Cellular ROS-related defense in the NAg^R^ and Ag^+T^ A. baumannii strains. (A) Fluorescent microscopy images of intracellular ROS (green) and dead cells (red) detected in the WT, NAg^R^, and Ag^+T^ strains, following their exposures to NAg and Ag^+^ (30 min). Also shown are the cell-only (untreated) and H_2_O_2_-treated samples (NA = not applicable). Scale bar = 10 μM. (B) Detection of cellular ROS (and dead cells) in the WT and NAg^R^ strains after 1 h of NAg exposure. (C) Quantitative analysis of the cellular ROS presence (as corrected total cell fluorescence, CTCF) and dead cells in the WT, NAg^R^, and Ag^+T^ strains, following 30 min of NAg, Ag^+^, and H_2_O_2_ exposures (left panel). The quantitative analysis of the ROS/dead cells, following 1 h of NAg exposure (for the WT and NAg^R^ strains) are also shown (right panel). For each strain, each of the exposure experiments was performed in three biological replicates, with five to six images being captured per replicate and 20 individual cells being analyzed per image. Asterisks indicate statistically significant differences in the cellular ROS level detected in the respective culture samples. *, *P* < 0.05; **, *P* < 0.01; ***, *P* < 0.001; ****, *P* < 0.0001.

The mutations that developed in silver-adapted A. baumannii could play a role in the defense mechanisms of the bacterium against NAg and Ag^+^. Herein, the defense traits were studied in the presence of silver. This was intended to reveal how the NAg^R^ and Ag^+T^ strains protected themselves from oxidative stress, one of the major toxicity paradigms of silver. For this study, the same silver-adapted isolates (used for the biofilm work) were examined in order to gain insights regarding the potential roles of the same mutations of interest in oxidative stress defense. To first confirm the ROS-associated antibacterial activity of NAg on A. baumannii, the WT strain was exposed to a sub-MIC dose of the nanoparticle (0.5× MIC at 0.5 μg/mL, 1 h exposure), and a higher cellular ROS presence was detected (presented as the corrected total cell fluorescence [CTCF], *P* < 0.001), relative to that of the untreated (cell-only) WT control (Fig. 3B and C). Apart from the earlier mentioned cell envelope targeting route, there are alternative pathways that can also promote silver-induced ROS generation in bacteria. Studies have shown that both NAg particulates and leached soluble silver can stimulate cellular ROS generation (34, 38, 73). Ag^+^, for instance, can attack iron-sulfur clusters that are present in many critical proteins, which releases Fenton-active ferrous (Fe^2+^) ions, which can then react with cellular H_2_O_2_ to generate highly reactive hydroxyl radicals (OH•) (23, 34). However, the ROS-generating mechanism of NAg particulates remains unclear at this stage.

When exposed to a similar NAg dose (0.5 μg/mL), there were fewer cellular ROS detected in the NAg^R^ strain, relative to the WT. The statistically significantly lower ROS level in the resistant strain was evident within just 30 min of NAg exposure (*P* < 0.05) (Fig. 3A and C), with the difference becoming even more significant after 1 h of exposure (*P* < 0.0001) (Fig. 3B, C). The findings are indicative of a NAg-induced, ROS-associated defense mechanism in the resistant strain, which may relate to the detected mutation in the CPS synthesis-related *rcsC* gene (Table 1). The increased CPS expression in A. baumannii has been linked to protection from antimicrobial targeting, and this includes antibiotics and oxygen radicals (83, 84). In fact, studies have reported an increased expression of the *cps* gene (which encodes CPS synthesis) as a result of *rcsC* mutation in E. coli (85, 86). Therefore, it is reasonable to suggest that the NAg-induced *rcsC* mutation detected in this study could correlate with the observed lower cellular ROS presence in the NAg^R^ strain. It is hypothesized that this links to the more compact occurrence of CPS structures on the NAg^R^ cell envelope, which hence limits the penetration of the toxic NAg particulates and leached soluble silver into the cell. The exposure of the NAg^R^ strain to ROS-generating H_2_O_2_ (30 min) also appeared to result in fewer cellular ROS in the resistant strain, compared to the WT, although this result was not statistically significant (Fig. 3A and C).

The cellular ROS studies with the Ag^+T^ strain revealed entirely different results. First, the ROS-associated antibacterial activity of Ag^+^ was indeed evident with the detection of a greater cellular ROS presence in the Ag^+^-exposed WT strain (0.5× MIC dose, 1 μg/mL, 30 min exposure, *P* < 0.0001), relative to that of the untreated WT control (Fig. 3C). With the Ag^+T^ strain, earlier studies have reported defense mechanisms in tolerant bacteria in response to (antibiotic-induced) cellular oxidative stress (58). Therefore, it was hypothesized that the Ag^+T^ strain would also exhibit cellular ROS-related defense mechanism(s), and these could possibly be associated with the evolved mutation in the antioxidant GSH synthesis gene *gshA*. However, as shown in Fig. 3A and C, the exposure of Ag^+T^ A. baumannii to Ag^+^ (1 μg/mL) resulted in a higher level of detected cellular ROS, relative to that of the WT strain, after just 30 min of exposure (*P* < 0.0001), which contrasts that seen with the NAg^R^ strain. This unexpected observation could perhaps relate to an enhancement in the respiratory activity of the tolerant strain, a known phenomenon in a number of microbial tolerance cases (87, 88). For instance, a study by McBee et al. reported a significantly higher cellular presence of the superoxide radical (O_2_•**^−^**) in antibiotic-tolerant Mycobacterium spp., and this appeared to result from enhanced respiration (as ROS is a natural byproduct of aerobic respiration) that is independent of antibiotic-induced oxidative stress (88, 89). Indeed, a higher level of cellular ROS was detected in the untreated Ag^+T^ strain, compared to the WT (*P* < 0.0001) (Fig. 3A and C), which might suggest that an increase in respiratory activity occurred independently of the silver-induced oxidative stress. However, it is still not clear at this stage whether the predicted increase in respiratory activity in the Ag^+T^ population correlates to any of the Ag^+^-induced SNPs (Table 1), as no studies have yet reported any link between this phenotype and any specific gene mutations. Increased respiratory activity has been indicated to occur stochastically in tolerant cells, with this phenotype being thought to assist the population in the resumption of normal growth upon the removal of antimicrobial pressure (87). A greater cellular ROS presence was also seen in the Ag^+T^ strain, following H_2_O_2_ treatment (30 min), relative to the WT strain (*P* < 0.0001) (Fig. 3A and C).

In summary, it is evident that the NAg^R^ strain developed a defense mechanism against nanoparticle-induced cellular oxidative stress. On the other hand, the Ag^+T^ strain exhibited a different cellular ROS-related response, which offers insights into the tolerant adaptation features of the bacterium. The tolerant strain appears to have physiologically transformed, displaying an apparent “overactive” respiration phenotype, which is a recognized characteristic of tolerant populations (87).

### Conclusions.

We report, for the first time, the ability of the Gram-negative opportunistic bacterial pathogen A. baumannii to establish a stable resistance phenotype to silver nanoparticles (NAg) while, notably, also developing tolerance to cationic silver (Ag^+^). The adaptation mechanisms are indicated to associate with stable physiological changes in order to protect the bacterium from silver toxicity. The NAg-resistant (NAg^R^) strain evolved mutations in genes that encode the protein subunits of the cell envelope organelles that mediate cell attachment to surfaces as well as a mutation in a cell envelope colonic acid capsular polysaccharide (CPS) synthesis-related gene. These mutations are suggested to correlate with the observed increase in biofilm growth in the resistant strain and to play a role in the suppression of oxidative stress (seen with the resistant strain). On the other hand, the “slower-to-kill” Ag^+^-tolerant (Ag^+T^) strain appeared to develop known tolerance features, including increased respiratory activity and changes in cell morphology. At this stage, however, it remains unclear as to how these features are linked to the detected mutations in the Ag^+T^ strain. Note that the present evolutionary adaptation studies were validated by the confirmation of the development of resistance to the model antibiotic nalidixic acid (Nx), with SNPs being detected in the target gene *gyrA* (reported involvement in Nx resistance). In closing, the findings highlight the risks of prolonged nanosilver use, presenting the need to overcome bacterial adaptations in order to ensure the successful long-term use of the important alternative antimicrobial agent. Further work is underway to elucidate the basis of the adaptation phenomena at the RNA and metabolite levels. The molecular work is anticipated to reveal other physiological changes and defense responses to silver toxicity, including any epigenetic (nonmutational) mechanisms. The upcoming work will also help to clarify the differences in the adaptation responses of A. baumannii to the nanoparticulate and ionic forms of silver in order to provide further insights into the roles of the gene mutations detected in the present study and to identify any compensatory mutations for fitness effects.

## MATERIALS AND METHODS

### Antibacterial agents.

Following the same procedure as described by Gunawan et al., (25) the silver nanoparticles (nanosilver; NAg) were synthesized into spherical particles (*d*_TEM_ ≈ 2 nm) via flame spray pyrolysis at the University of New South Wales (UNSW), with Ag_2_O being finely dispersed onto inert 30 nm TiO_2_ (Ag = 6.60 wt%). The particles were sterilized via gamma irradiation (Co-60) for 1 h at an approximately 6 Gy/min dose at the Australian Nuclear Science Technology Organisation (ANSTO). Nanoparticle suspensions (in culture media) were ultrasonicated (20 s, 50% amplitude, Vibra-cell, Sonics & Materials, Inc.) prior to each bacterial exposure experiment. Silver nitrate (AgNO_3_) (Merck & Co. Inc.) was used as the source of ionic silver (Ag^+^). Nalidixic acid (Nx) (Sigma-Aldrich) was used as the model conventional antibiotic. This antibiotic drug was selected, following a preliminary disc diffusion assay, in order to determine the susceptibility profile of the model A. baumannii strain ATCC 19606 to different antibiotic classes. The wild-type (WT) strain was found to be resistant to several antibiotics (≤6 mm zone of inhibition as classified under the Clinical and Laboratory Standards Institute (CLSI) guidelines), including ampicillin (penicillins), gentamicin, streptomycin, neomycin, netilmicin (aminoglycosides), ceftriaxone (cephalosporins), chloramphenicol (chloramphenicols), spectinomycin (aminocyclitols), sulfonamide (sulfonamides), and trimethoprim (antifolates). The WT strain was found to be susceptible to Nx (fluoroquinolones) but is known to develop resistance to the drug via a verifiable point mutation in the gene *gyrA* (48), hence it serving as the reference point in the validation of the present evolutionary adaptation study.

### Bacterial culture and growth conditions.

The A. baumannii model organism strain ATCC 19606, isolated from a urine sample in 1948 by Schaub and Hauber (90), was used throughout this study. A frozen (−80°C) glycerol-culture stock of wild-type (WT) ATCC 19606 was streaked onto a cation-adjusted Mueller-Hinton agar (CAMHA) plate (BD, Australia) and incubated at 37°C for 16 to 18 h. For each antibacterial exposure experiment, an overnight broth culture of A. baumannii was prepared in cation-adjusted Mueller-Hinton broth (CAMHB) (BD, Australia) and incubated for 16 to 18 h at 37°C with shaking at 250 rpm. Overnight cultures were diluted 1:100 in fresh CAMHB to approximately 2.4 × 10^8^ CFU/mL and were further incubated for 2 h (37°C, 250 rpm) to precondition the culture prior to experimentation. This process reestablishes the active log-phase of bacterial growth (91).

### MIC determination.

The MICs were determined using the standard broth microdilution technique, as previously described (92), and they were interpreted using the Clinical and Laboratory Standards Institute (CLSI) breakpoints (93). WT A. baumannii was exposed to increasing concentrations of NAg, Ag^+^, and Nx in a 96-well microtiter plate (BD Falcon, USA). The antibacterial experiments were performed at 37°C for 24 h in dark conditions so as to render the TiO_2_ support photocatalytically inactive for the NAg particulates (25) and to prevent the reduction of Ag^+^ to metallic silver (Ag^0^) (94). The lowest dose of each agent with no visible growth was reported as the MIC. Note that for the NAg MIC, the presence of the particulates in the growth media affects the turbidity of the exposure system. Thus, the assigned MIC of NAg was confirmed through agar plate streaking to ensure that no bacterial colony growth had occurred.

### Adaptation responses via sequential passaging.

WT A. baumannii was sequentially passaged (subcultured every 24 h) in the presence of increasing concentrations of NAg, Ag^+^, and Nx in a 24-well microtiter plate (BD Falcon, USA) at 37°C and 150 rpm, using 1,000 μL working volumes. On day 1, ATCC 19606 was exposed to 0.25×, 0.5×, 1×, and 2× MIC doses of the respective agent. The highest dose of each agent with observable growth after 24 h was recorded. For subculturing, 100 μL of the surviving culture (from wells containing the highest agent dose with observable growth) were transferred (1:10 dilution) into a new exposure system with progressively increasing MIC-fold ranges of each agent. A cell count of approximately 1.7 × 10^8^ CFU/mL was present at the start of each passage step. This process was repeated for 30 days with approximately 150 total cell generations occurring. The sequential passaging experiments were performed in two biological replicates (each with three technical triplicates) and included cell-only and media-only controls. The cell-only control (containing no antibacterial agent) was used to rule out any development of background “random” adaptation/mutations. Samples of each technical triplicate from the biological passage replicates were stored in 80% glycerol in Cryotubes (Sarstedt, Germany) at −80°C. To confirm the development of stable resistance traits, each replicate sample was streaked onto CAMHA plates that contained the respective antibacterial agent (minimum 2× MIC dose) to select for “adapted” colonies. Single isolates (one colony) from each technical replicate (across the biological passage replicates; *n* = 6) were grown in antibacterial-free CAMHB for three days (1:100 subculturing every 24 h, 37°C, 250 rpm) to exclude cell populations with transient adaptation traits, and they were then tested for (at least) a 2-fold increase in MIC (46). Note again that for the NAg MIC assay, the determined MIC was confirmed via agar plate streaking to ensure that no bacterial growth had occurred. This included the NAg MICs in the cross-adaptation study. A cross-adaptation study was carried out to determine whether there was a change in the Ag^+^ and Nx MICs of the NAg-passaged strain, the NAg and Nx MICs for the Ag^+^-passaged strain, and the NAg and Ag^+^ MICs for the Nx-passaged strain.

### Time-kill assay.

Overnight, preconditioned cultures of WT and Ag^+^-passaged A. baumannii were exposed to a 1.5× MIC dose of Ag^+^ in 50 mL of CAMHB at 37°C and 200 rpm for 3 h. Cultures were sampled (1 mL) every 30 min (including at time 0 min). The samples were serially diluted in phosphate-buffered saline (PBS), spread on CAMHA plates, and incubated at 37°C for 24 h. Plates with 30 to 300 surviving colonies (at the respective dilution factor) were counted, and the fraction of surviving colonies (in colony forming units/mL [CFU per mL]) per time point were plotted, relative to the CFU population at time 0. Each assay was performed in at least two biological replicates, and each Ag^+^-passaged biological replicate was included in the study. Untreated cell-only controls of the WT and Ag^+^-passage strains were also included.

### Growth rate assay.

Overnight, preconditioned cultures of WT, NAg-passaged, and Ag^+^-passaged A. baumannii were diluted to an OD_600_ of 0.05 and incubated at 37°C and 150 rpm for 6 h in a 96-well plate. Each hour, spectrophotometric readings were performed to record the OD_600_ of each culture. Each biological passage replicate of the NAg- and Ag^+^-passaged A. baumannii was included. The OD_600_ values are reported in log_10_ format.

### Whole-genome extraction, sequencing, and assembly.

Prior to the whole-genome analysis, the passage replicate cultures were first subjected to antimicrobial-free “posttreatment” to exclude cell populations with transient adaptation traits. The passaged cultures (six individual replicates for each of the NAg, Ag^+^, and Nx passaging experiments) were grown on selective CAMHA plates (containing the respective antibacterial agent, minimum 2× MIC dose) to isolate the “adapted” colonies. These adapted colonies (six isolated colonies from each of the NAg-, Ag^+^-, and Nx-passaged experiments; each colony represents the replicate cultures of the passaging experiments) were then grown in antibacterial-free CAMHB media for 3 days (1:100 subculturing every 24 h, 37°C, 250 rpm). DNA extraction was carried out on the resulting cultures (six adapted isolates from each antimicrobial agent, including the passaged cell-only control) using a DNeasy Blood and Tissue Spin-column Kit (Qiagen, Germany). The genomic DNA concentration and quality were assessed using a Quant-iT PicoGreen dsDNA Assay Kit (Invitrogen, USA) and agarose gel electrophoresis. The whole-genomes were sequenced at Sequencing @ UTS (University of Technology Sydney, Australia), and the library preparation was done using an adapted Nextera Flex protocol (Hackflex) (95). Briefly, tagmented DNA was amplified using the facility’s custom designed i7 and i5 barcodes that involve 12 cycles of PCR, and this was followed by collation into a library pool, which was then cleaned using SPRIselect beads (Beckman Coulter, USA). The final pool was sequenced using an Illumina Novaseq S4 flow cell (2 × 150 bp) at Novogene (Singapore). The DNA sequence data were assembled in draft genome sequences using Shovill v1.0.9 (https://github.com/tseemann/shovill).

Comparative whole-genome alignment was carried out using Snippy v4.6.0, with WT ATCC 19606 (GenBank accession number CP045110.1) being used as the reference genome. The manual curation of variant call files was performed to identify the genomic locations of the single nucleotide polymorphisms in the passaged strains and the metabolite/protein products of the (mutated) genes. Furthermore, the draft genomes of the WT strain were checked, and no new genomic changes due to mobile genetic element movement, were detected. Unknown gene identities were confirmed through comparative analyses of other annotated genome sequences of ATCC 19606 (GenBank accession numbers CP046654.1 and CP059040.1) that were determined by different research groups.

Sanger sequencing was performed (by the Australian Genome Research Facility) to confirm the presence of the detected single nucleotide mutations. PCR was used to generate amplicons of the target genes using the primers (Integrated DNA Technologies [IDT], USA) listed in Table S3. PCRs (35 amplification cycles) were carried out with a 60 to 62°C, 30 to 35 s annealing step and a 72°C, 1 min extension step. The quality of the amplicons was assessed using gel electrophoresis. The PCR products were cleaned using either a sodium acetate (3 M) + 100% ethanol precipitation method or gel extraction using a QIAquick Gel Extraction Kit (Qiagen, Germany).

### Biofilm fluorescence staining, imaging, and biomass quantification.

For biofilm growth, overnight cultures of WT, NAg-passaged, and Ag^+^-passaged strains were diluted 1:100 in fresh CAMHB, inoculated into a FluoroDish (World Precision Instruments, USA), and incubated for 24 h at 37°C. Following the incubation, the cultures were resuspended in 1 mL PBS. Biomasses were stained with live/dead SYTO-9/propidium iodide (PI) dyes to working concentrations of 5 μM and 30 μM, respectively (Thermo Fisher Scientific, USA), for 30 min at room temperature in dark conditions. The stained biomasses were washed with PBS (2×), and 80% glycerol was then added to each dish.

Fluorescent imaging was performed using a DeltaVision (DV) Elite deconvolution fluorescence microscope (GE Healthcare, USA), with 475/28 nm excitation and 523/48 nm (green) emission for SYTO-9 (stained all cells) and 632/22 nm excitation and 679/34 nm (red) emission for PI (stained cells with compromised cytoplasmic membranes, indicative of dead cells). All of the sectional Z-stack images were captured using the DV elite SoftWoRx program and deconvolved. Phase contrast images of planktonic (nonbiofilm) cultures of each strain were also captured using the DV Elite microscope. The deconvolved biofilm images were analyzed using Imaris software v9.6.0 (Oxford Instruments, UK) to determine the biofilm biomass (μm^3^/μm^2^; μm^3^ = biomass volume, μm^2^ = surface coverage). Raw (nondeconvolved) images were used to provide visualizations of the biofilm biomass/cell morphology, and they are presented as single plane 3D slices. A statistical analysis was performed in Prism (GraphPad, USA) using an unpaired Student’s *t* test (with Welch’s correction). A *P* value of <0.05 was regarded as indicative of a statistically significant result.

### Reactive oxygen species generation assay.

For the cellular ROS staining, overnight, preconditioned cultures of WT, NAg-passaged, and Ag^+^-passaged strains were first suspended in 1 mL saline (8 g/L NaCl + 0.2 g/L KCl) and stained with H_2_DCFDA (10 μM working concentration, Invitrogen, USA) for 45 min at room temperature under dark conditions. The stained cultures were then washed, resuspended in 1 mL saline, and treated with a 0.5× MIC doses of NAg and Ag^+^, respectively, for 30 min and 60 min at 37°C and 200 rpm. For the dead cell staining, treated cultures were resuspended in 1 mL saline stained with PI (30 μM working concentration, Invitrogen, USA) for 5 min at room temperature under dark conditions. Finally, the cultures were washed and resuspended in 100 μL saline, and 3 μL of the sample were loaded onto a glass slide with a Gene Frame (Thermo Fisher Scientific, USA) that contained a 2% agarose gel pad. A positive control of hydrogen peroxide-treated cells (50 mM) and an untreated cell-only control were included.

Fluorescent imaging was performed using the DV Elite microscope (GE Healthcare, USA), with 475/28 nm excitation and 523/48 nm (green) emission for H_2_DCFDA. For PI, the excitation/emission wavelengths were as listed above. Images were captured using the DV Elite SoftWoRx program and were deconvolved. The quantitative analysis was performed using the open-source image processor Fiji (ImageJ, USA). The fluorescence intensity (which is correlated with the cellular ROS level) was quantified by calculating the corrected total cell fluorescence (CTCF). Briefly, a selected cell was outlined using the ROI tool, from which the “area”, “integrated density”, and “mean gray value” parameters were measured, along with the background (i.e., area with no fluorescence). The CTCF was calculated using the following formula.

Integrated density–(area of selected cell×mean fluorescence of background readings)

20 (*n* = 20) cells and 10 (*n* = 10) background readings were assigned per image for each replicate treatment (5 or 6 images) per strain. The dead cells were manually counted and were recorded as a percentage of the total number of cells per image. The statistical analysis was performed in Prism (GraphPad, USA). An unpaired Student’s *t* test (with Welch’s correction) was used.A *P* value of <0.05 was regarded as indicative of a statistically significant result.

### Data availability.

The draft genome sequence data for the mutant ATCC 19606 strains that were determined in this study have been deposited in the GenBank/EMBL/DDBJ database and are publicly available under the GenBank BioProject accession number PRJNA914845.
