# Supplementary material for: Development of Nanoparticle Adaptation Phenomena in Acinetobacter baumannii: Physiological Change and Defense Response
Source: Microbiol Spectr. 2023 Jan 10;11(1):e02857-22. doi: 10.1128/spectrum.02857-22 (PMC9927149; doi:10.1128/spectrum.02857-22)
Supplement: Supplemental file 1 — Supplemental material. Download spectrum.02857-22-s0001.pdf, PDF file, 0.7 MB [file spectrum.02857-22-s0001.pdf]

Supplementary Information

Development of Nanoparticle Adaptation Phenomena in *Acinetobacter baumannii*: Physiological Change and Defence Response

Oliver McNeilly[a], *Riti Mann*[a], *Max Laurence Cummins*[ab], *Steven P. Djordjevic*[ab], *Mehrad Hamidian**[a], *Cindy Gunawan**[ac]

[a] Australian Institute for Microbiology and Infection, University of Technology Sydney, Broadway, NSW 2007, Australia

[b] Australian Centre for Genomic Epidemiological Microbiology, University of Technology Sydney, Broadway, NSW 2007, Australia

[c] School of Chemical Engineering, University of New South Wales, Sydney, NSW 2052, Australia

***** Correspondence: mehrad.hamidian@uts.edu.au, cindy.gunawan@uts.edu.au

A **Prolonged antibacterial exposure via sequential passaging**

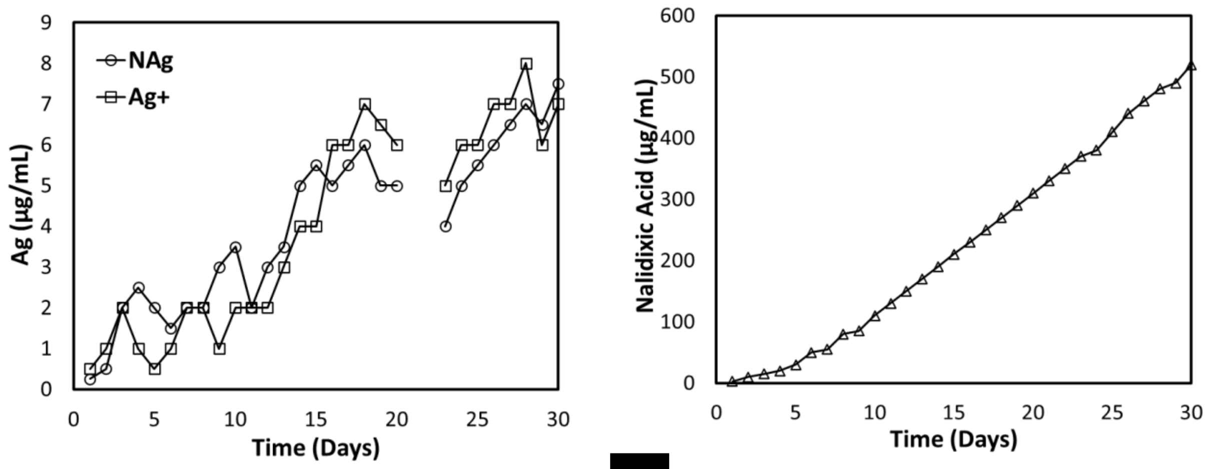

B **Evolved stable adaptation phenotypes**

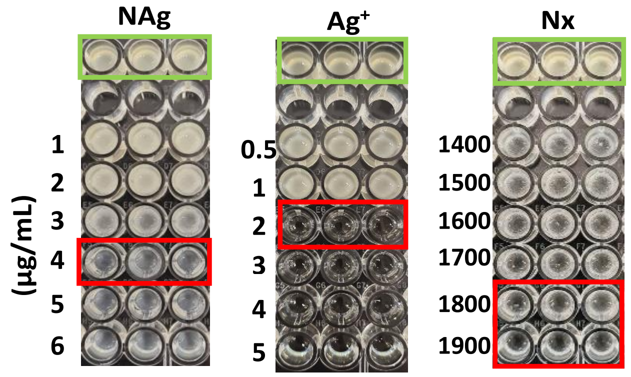

Antibacterial agent	MIC (µg/mL)
NAg	4
Ag^{+}	2
Nx	1800-1900

C

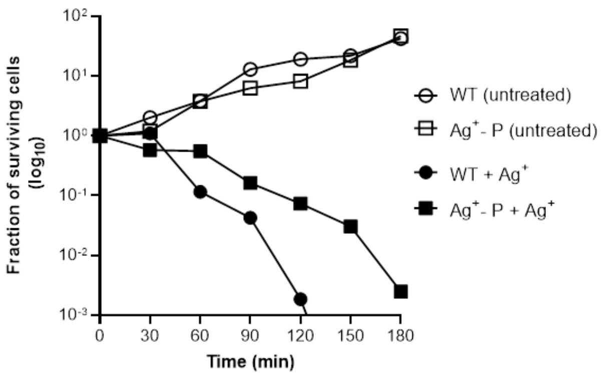

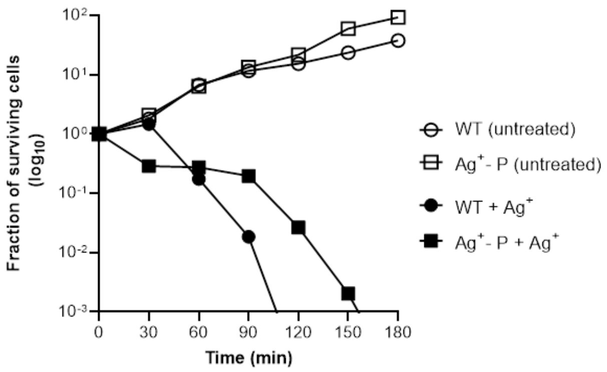

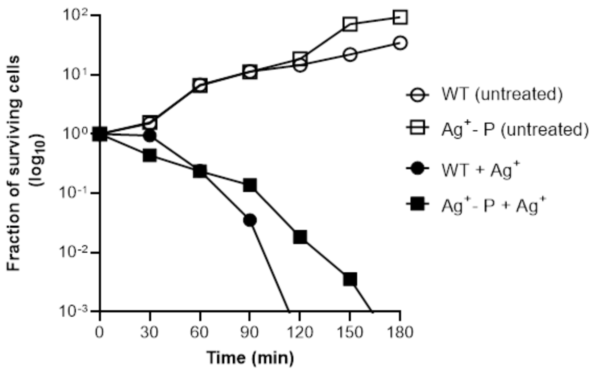

Strain	MDK_{99}
WT *A. baumannii*	~ 90-105 mins
Ag^{+}-passaged *A. baumannii*	~ 135-165 mins

Fig. S1. Evolution of adaptation phenotypes to NAg, Ag^+, and Nx in *A. baumannii.* (**A**) Second biological replicate of sequentially passaged *A. baumannii* in the presence of progressively increasing NAg, Ag^+, and Nx concentrations over 30 days. (**B**) NAg, Ag^+, and Nx minimum inhibitory concentration (MIC) assessments for the second biological replicate of sequentially passaged *A. baumannii* (green outline = cell-only control; red outline = MIC point). (**C**) Killing kinetics of WT and Ag^+-passaged (Ag^+-P) *A. baumannii* when exposed to 1.5 x Ag^+ MIC dosage, for determination of the minimum duration of killing 99% (MDK_{99}) of the cell populations. The plots show the second technical replicate of the first biological passaged replicate (for the Ag^+-P strain) (top left), and the first technical replicate (lower left) and second technical replicate (right) of the second biological passaged replicate (for the Ag^+-P strain).

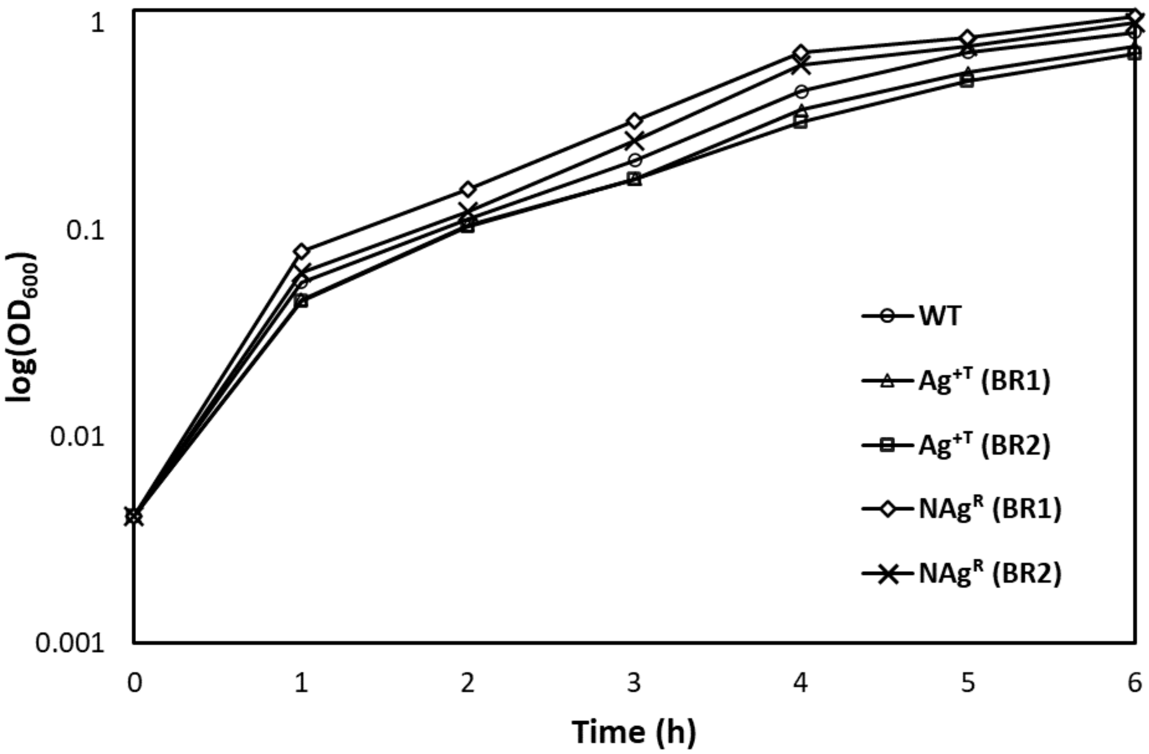

Fig. S2. Growth of WT, NAg-resistant (NAg^{R}), and Ag^{+}-tolerant (Ag^{+T}) *A. baumannii.* Shown are the 6 h growth profiles of the NAg^{R} and Ag^{+T} strains from each biological passage replicate (BR1 and BR2). Note the initial OD_{600} (optical density at 600 nm) of 0.05 for each culture system.

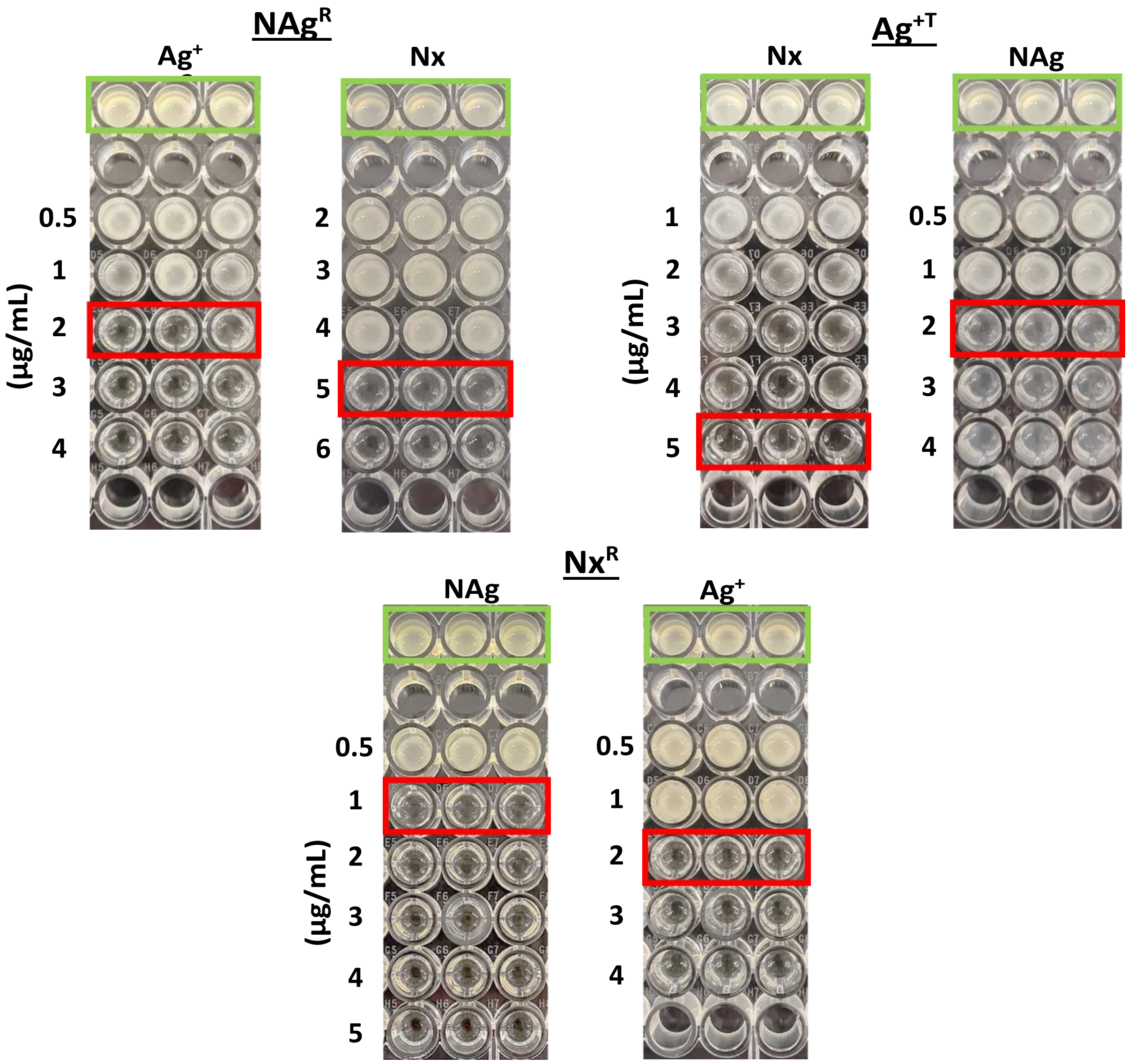

Strain	Antibacterial agent		
	NAg	Ag^+	Nx
NAg^R	-	S	S
Ag^{+T}	S	-	S
Nx^R	S	S	-

Fig. S3. Comparative cross-adaptation study between the NAg-resistant (NAg^R), Ag^+-tolerant (Ag^{+T}), and Nx-resistant (Nx^R) strains. Each adapted strain was tested for an increase in MIC between to their respective non-passaged agent to determine if a cross-adaptation effect had developed during the sequential passage experiment. Green outline = cell-only control; red outline = MIC point. The table highlights the susceptibility profile (sensitive = S, resistant = R) of each strain following MIC testing with the respective agents. Each biological passage replicate was tested in this cross-adaptation study and produced identical results. Only the results of the one biological replicate are shown.

Table S1. 'Background' gene mutations detected in the cell-only passaged control culture (containing no antimicrobial)

Mutation	Genome pos.[1]	AA change	Gene	Locus tag	Protein product
SNP (G > A)	787051	Gly195Ser	*rstA*	FQU82_00751	Transcriptional regulator protein RstA
Del (AT > A)	1438692	Phe444fs [2]	*actP*	FQU82_01385	Copper-transporting P-type ATPase
SNP (T > C)	1879011	Gln222Arg	*hrtE*	FQU82_01809	Outer membrane usher protein HtrE
Ins (C > CATTAAAAAT)	2090006	Asn90dup [3]	*yjiE*	FQU82_02014	HTH-type transcriptional regulator YjiE
SNP (G > A)	2156407	Val67Ile	*sasA*	82_02072	Adaptive-response sensory-kinase SasA
SNP (G > A)	2665349	Ser58Leu	*csuE*	82_02549	Csu pili tip adhesin CsuE
SNP (C > A)	2980241	Trp273Leu	*gltR*	82_02866	HTH-type transcriptional regulator GltR
SNP (G > A)	3166695	Ala320Val	*blp1*	82_03028	Blp1

[1] Position of gene mutation in the *A. baumannii* genome. [2] fs = frameshift. [3] dup = duplication

Table S2. The complete list of gene mutations detected in the Nx^R strain. Mutations to *gyrA* gene (**Table 1**) and random 'background' mutations (**Table S1**) have been excluded.

Mutation	Genome pos. [1]	AA change	Gene	Locus tag	Protein product
SUB (C >T)	620945	Ala165Thr	*macA*	FQU82_05595	Macrolide export protein MacA
SUB (A > G)	658605	Leu598Pro	*rcsC* [2]	FQU82_00629	Sensor histidine kinase RcsC
SUB (A > G)	659115	Ile428Thr	*rcsC* [2]	FQU82_00629	Sensor histidine kinase RcsC
SUB (C > T)	708108	Cys275Tyr	*gigA*	FQU82_00676	RsbU family protein phosphatase GigA
SUB (C > T)	708291	Leu214Pro	*gigA*	FQU82_00676	RsbU family protein phosphatase GigA
SUB (A > G)	718107	-	INTERGENIC [3]	-	-
SUB (C > T)	1114223	Ala9Val	-	FQU82_01057	Hypothetical Protein [4]
SUB (C > T)	1118624	-	INTERGENIC [3]	-	-
SUB (A > G)	1338851	Ala330Ala	nk [5]	FQU82_01287	ATP-binding protein
SUB (C > T)	1497310	Arg1782Cys	nk [5]	FQU82_01459	GNAT family N-acetyltransferase
INS (T > TG)	1526729	Leu104fs	*yigZ*	FQU82_01486	IMPACT family member YigZ
SUB (G > A)	1531871	His815Tyr	*mutS*	FQU82_01493	DNA mismatch repair protein MutS
SUB (C > T)	1726410	Asp352Asp	nk [5]	FQU82_01662	DCAP-like protein
INS (G > GC)	1776690	His256fs	*mdcE*	FQU82_01714	Biotin-independent malonate decarboxylase
SUB (C > T)	1889732	Met48Ile	-	FQU82_01822	Hypotheical Protein [4]
SUB (C > T)	1974783	Ala134Ala	nk [5]	FQU82_01906	Glutathione S-transferase
SUB (A > G)	2046714	Leu339Leu	*metZ*	FQU82_01973	O-succinylhomoserine sulfhydrylase
SUB (C > T)	2153492	Val204Met	*acrB*	FQU82_02069	Multidrug efflux pump subunit AcrB
SUB (C > A)	2154730	Gly197Cys	*mexA*	FQU82_02070	Multidrug resistance protein MexA
DEL (GA > A)	2156077	Lys217fs	*srrA*	FQU82_02071	Transcriptional regulatory protein SrrA
DEL (GA > A)	2360141	Lys515fs	*cydA*	FQU82_02258	Cytochrome bd upiquinol oxidase subunit 1
INS (A > AC)	2384649	Ile228fs	nk [5]	FQU82_02286	EAL domain-containing protein
DEL (TA > T)	2517316	-	INTERGENIC [3]	-	-
INS (C > CA)	2572117	Ala484fs	*kdpD*	FQU82_02458	Sensor protein KdpD
SUB (G > A)	2622129	Gly110Arg		FQU82_02505	Ribosomal protein L11 methyltransferase
SUB (T > C)	2744324	-	INTERGENIC [3]	-	-
DEL (CA > C)	2990495	-	INTERGENIC [3]	-	-
SUB (T > C)	3005539	Tyr294Tyr		FQU82_02886	Nitronate monooxygenase
SUB (T > C)	3017346	Leu302Leu	*uvrB*	FQU82_02897	UvrABC system protein B
DEL (CA > C)	3121018	Lys146fs	nk [5]	FQU82_02998	Tim44 domain-containing protein
SUB (G > A)	3191294	Ala667Thr	*carB*	FQU82_03583	Carbamoyl-phosphate synthase large chain
INS (A > AG)	3363303	-	INTERGENIC [3]	-	-
INS (G > GA)	3659282	-	INTERGENIC [3]	-	-
SUB (G > A)	3666751	-	INTERGENIC [3]	-	-
SUB (T > G)	3751924	Ser183Arg	*rhaS*	FQU82_03583	HTH-type transcriptional activator RhaS

[1] Position of gene in *A. baumannii* genome. [2] Mutation to gene(s) also detected in NAg^R strain. [3] Mutation occurred in the intergenic region/s of genome (no open-reading frame present). [4] No putative function allocated for gene/protein. [5] nk = gene name not known

Table S3. List of primers used for validation of mutations detected in the NAg^R strain. See the **Experimental Section** for the PCR amplification method.

Gene	Product size (bp)	Forward primer (5' → 3')	Reverse primer (3' → 5')	Annealing temperature (°C)
rcsC	421	GAAAACCGATTTTGCCTGAT	CCCTGTCTGGCTCTTGATTG	60
smf1-2	770	AGCTTGGACAAGTGCTGGTT	GCTGCTCTTTCGGTTGTAGG	60
csuB	920	GCCAGACGGTTTGTAGGTGT	AGTAAATGCGGGTGAAATCG	63
